# The Effect of Pressure on Halogen Bonding in 4-Iodobenzonitrile

**DOI:** 10.3390/molecules24102018

**Published:** 2019-05-27

**Authors:** Nico Giordano, Sergejs Afanasjevs, Christine M. Beavers, Claire L. Hobday, Konstantin V. Kamenev, Earl F. O’Bannon, Javier Ruiz-Fuertes, Simon J. Teat, Rafael Valiente, Simon Parsons

**Affiliations:** 1 Centre for Science at Extreme Conditions and EaStCHEM School of Chemistry, The University of Edinburgh, King’s Buildings, West Mains Road, Edinburgh, Scotland EH9 3FD, UK; Nico.Giordano@ed.ac.uk (N.G.); Claire.Hobday@ed.ac.uk (C.L.H.); 2 Advanced Light Source, 1 Cyclotron Road, Lawrence Berkeley National Laboratory, Berkeley, CA 94720, USA; obannon2@llnl.gov; 3 Centre for Science at Extreme Conditions and School of Engineering, The University of Edinburgh, King’s Buildings, West Mains Road, Edinburgh, Scotland EH9 3FD, UK; S.Afanasjevs@ed.ac.uk (S.A.); k.kamenev@ed.ac.uk (K.V.K.); 4 Department of Earth & Planetary Sciences, University of California, Santa Cruz, 1156 High Street Santa Cruz, CA 95064, USA; 5 Present address: Diamond Light Source, STFC Rutherford Appleton Laboratory, Harwell Science and Innovation Campus, Harwell Oxford, Didcot OX11 0QX, UK; 6 Present address: Physical and Life Sciences, Physics Division, Lawrence Livermore National Laboratory, Livermore, CA 94551, USA; 7 Dpto. DCITIMAC, Facultad de Ciencias, Universidad de Cantabria, 39005 Santander, Spain; javier.ruizfuertes@unican.es; 8 Dpto. Física Aplicada, Facultad de Ciencias, Universidad de Cantabria-IDIVAL, 39005 Santander, Spain; rafael.valiente@unican.es

**Keywords:** high pressure, halogen bonding, intermolecular interactions

## Abstract

The crystal structure of 4-iodobenzonitrile, which is monoclinic (space group *I*2/*a*) under ambient conditions, contains chains of molecules linked through C≡N···I halogen-bonds. The chains interact through CH···I, CH···N and π-stacking contacts. The crystal structure remains in the same phase up to 5.0 GPa, the *b* axis compressing by 3.3%, and the *a* and *c* axes by 12.3 and 10.9 %. Since the chains are exactly aligned with the crystallographic *b* axis these data characterise the compressibility of the I···N interaction relative to the inter-chain interactions, and indicate that the halogen bond is the most robust intermolecular interaction in the structure, shortening from 3.168(4) at ambient pressure to 2.840(1) Å at 5.0 GPa. The π∙∙∙π contacts are most sensitive to pressure, and in one case the perpendicular stacking distance shortens from 3.6420(8) to 3.139(4) Å. Packing energy calculations (PIXEL) indicate that the π∙∙∙π interactions have been distorted into a destabilising region of their potentials at 5.0 GPa. The structure undergoes a transition to a triclinic ($P\bar{1}$) phase at 5.5 GPa. Over the course of the transition, the initially colourless and transparent crystal darkens on account of formation of microscopic cracks. The resistance drops by 10% and the optical transmittance drops by almost two orders of magnitude. The I···N bond increases in length to 2.928(10) Å and become less linear [<C−I∙∙∙N = 166.2(5)°]; the energy stabilises by 2.5 kJ mol^−1^ and the mixed C-I/I..N stretching frequency observed by Raman spectroscopy increases from 249 to 252 cm^−1^. The driving force of the transition is shown to be relief of strain built-up in the π∙∙∙π interactions rather than minimisation of the molar volume. The triclinic phase persists up to 8.1 GPa.

## 1. Introduction

Hydrogen bonded solids were amongst the first organic systems of any complexity to be studied at high pressure. One of the first studies concerned hydrogen-bonding in oxalic acid [1], and a substantial body of work now exists which demonstrates that hydrogen-bonded systems are highly sensitive to pressure, exhibiting extensive phase diversity on compression, the amino acids having been studied particularly thoroughly [2,3,4]. A notable example of the effect of pressure is in migration of the proton positions between the hydrogen-bond donor-acceptor sites in the oxalic acid dihydrate and in cyclohexane-1,3-dione [5,6,7].

Other similar classes of interaction, such as the analogous halogen bond, R-X∙∙∙D (where R = organic group, X = halogen, D = nucleophilic site) [8], which have the potential to be highly sensitive to pressure, have been more lightly investigated. Of the 2715 high pressure structures deposited in the Cambridge Structural Database (CSD) (v5.40, November 2018), only 41 exhibit halogen bonding. Interest in the area mainly concerns the role of halogen bonding in polymorphism [9,10] and in charge transfer properties of materials [11,12]. In crystal engineering the halogen bond has a propensity to be highly directional, and it has been used to direct chain formation in polymerisation reactions at high pressure [13]. The σ -hole (a region of positive electrostatic potential on the halogen atom) [14] forms along the axis of the covalent bond and directs intermolecular interactions with the nucleophile to be linear (R-X∙∙∙D = 180°). The degree of atomic separation and interaction energies are affected by the electrostatic potential of the sigma hole, which can be tuned by changing the electron withdrawing affinity of the R-group, or by substituting the halogen atom for another which leads to a plethora of compounds with halogen bonding [15,16,17].

Halogen bond interactions between N and I have been identified as a potential candidates for high-pressure studies as they have already been shown to be sensitive to variations in temperature [18]. The high polarizability of iodine makes its bonding highly flexible. For example, elemental iodine, which at ambient pressure contains semi-conducting planes of donor-acceptor interactions (I∙∙∙I = 3.50 and 3.97 Å, where 2× the van der Waals radius of I is 4.08 Å [19]), transforms to a metal at 16 GPa [20]. In the context of other metallisation transitions in the main-group elements, this is quite a modest pressure. Very recently compression beyond 10 GPa of the organic polyiodide salt tetraethylammonium diiodine triiodide has been shown to lead to an insulating-to-semiconducting transformation driven by donor-acceptor bond formation between iodine molecules and tri-iodide anions [21].

**Scheme 1:**
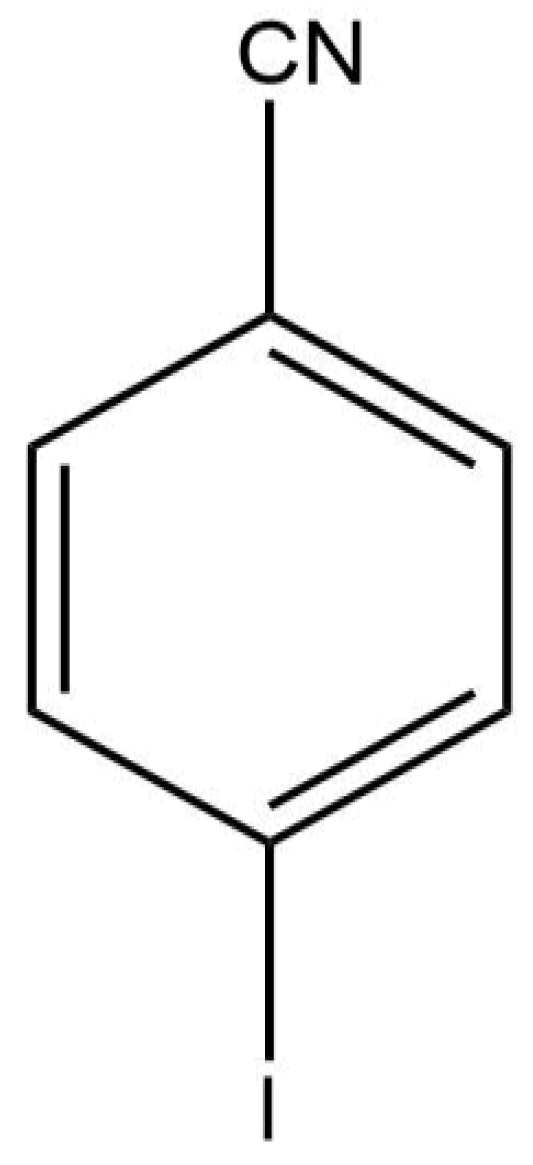


The I∙∙∙N intermolecular distance in 4-iodobenzonitrile (Scheme 1) is 3.168(4) Å which is substantially shorter than the sum of the van der Waals radii (3.70 Å) [19]. In this paper we describe the effects of pressure on this interaction up to 8.1 GPa with the aim of characterising its sensitivity to compression.

## 2. Results and Discussion 

### 2.1. Ambient Pressure Structure and the First Coordination Sphere

4-Iodobenzonitrile crystallises in phase I at ambient conditions from dichloromethane or a mixture of methanol and ethanol and at high pressure (0.6 GPa) from methanol-ethanol. The crystal structure of 4-iodobenzonitrile has been determined previously by Schlemper and Britton in 1965 [22], and subsequently by Desiraju and Harlow in 1989 [23]. The structure has also been compared with those of other iodobenzene derivatives by Merz [24]. The space group is *I*2/*a*, with the four molecules in the unit cell residing on crystallographic two-fold axes. The intermolecular contacts are listed in Table 1, where contacts are labelled A-L in order of energy as calculated by PIXEL. 

**Figure 1:**
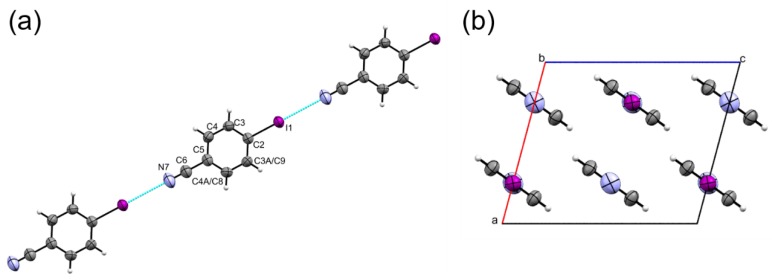
(**a**) ORTEP style plot of 4-iodobenzonitrile and the numbering scheme used in this study. The atom numbering scheme in phase II is the same as that of phase I but the symmetry generated C3 and C4 become C9 and C8, respectively. Ellipsoids are drawn at the 50 % probability level. (**b**) Molecular chains formed along **b** surrounded by six neighbouring chains.

Molecules related by unit cell translations along **b** (molecules G and H in Table 1) are linked by I···N halogen bonding interactions into an exactly linear chain, with a contact distance of 3.168(4) Å and a total intermolecular energy of −10.8 kJ mol^−1^ (Figure 1a). Note that this is not the strongest interaction in the structure. For comparison, Carlucci and Gavezzotti reported a dimer energy of −14.3 kJ mol^−1^ for the interaction involving the I∙∙∙N contact (at 3.1 Å) in the pyrazine-iodobenzene cocrystal [15].

**Figure 2:**
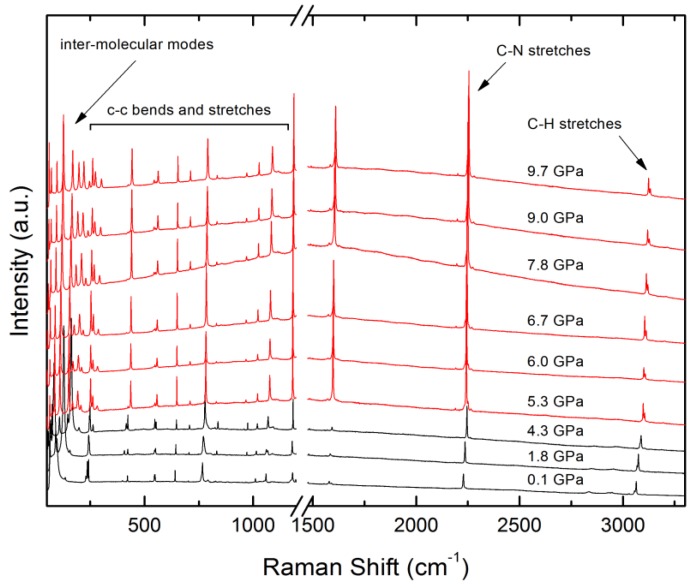
Raman spectra collected on compression of 4-iodobenzonitrile over the entire spectral range studied. Phase I spectra are shown in black, and phase II spectra in red.

**Figure 3:**
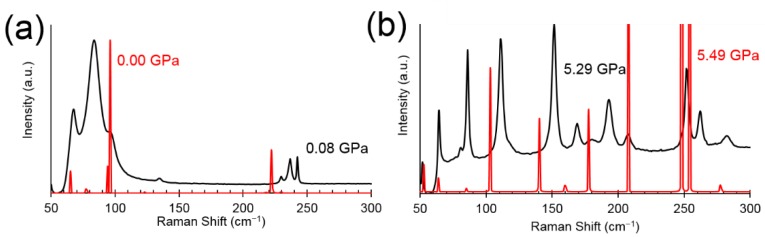
Comparison of the lattice phonon regions in the Raman spectrum of 4-iodobenzobitrile. (**a**) Phase I at ca. ambient pressure. Black: measured at 0.08 GPa in a diamond anvil cell, red: calculated form the ambient-pressure crystal structure by periodic DFT. (**b**) Phase II at *ca* 5.5 GPa. Black: measured at 5.29 GPa, red: calculated from the crystal structure at 5.49 GPa.

The Raman spectra of 4-iodobenzonitrile are shown as a function of pressure in Figure 2 with an expansion of the lattice mode region at ambient pressure in Figure 3a, where the experimentally measured spectra are in black with the positions and intensities of the modes calculated by periodic DFT in red. The DFT frequencies, which are unscaled, reproduce the experimental values within 20 cm^−1^, while the intensities are less reliably reproduced, as is usual. The DFT results indicate that the strong bands between 50 and 100 cm^−1^ are whole-molecule rocking motions. The calculations also indicate that there are three Raman active modes at 229 (intensity 8 Å^4^ amu^−1^), 233 (186) and 241 (16) cm^−1^, which agrees with the three observed modes at 230, 237 and 243 cm^−1^). Of these, the central band has A_g_ symmetry in the C_2h_ point group of the crystal lattice and can be described as a mixture of C-I and I∙∙∙N stretching modes; the other two modes have B_g_ symmetry. There are two molecules in the primitive setting of the unit cell and the corresponding anti-symmetric mode is only infra-red active (calc. 235 cm^−1^, A_u_ symmetry). 

Each halogen-bonded chain in the crystal structure is surrounded by six others, with offset π∙∙∙π stacking and other dispersion-based interactions between them which range in energy from −6.8 to −12.6 kJ mol^−1^ (Figure 1b). The shortest contacts between the chains are between C4H4∙∙∙I1 and C4H4∙∙∙N7 at ca. 3.3 Å in molecules A to F which have total molecule-molecule energies of −12.6 and −11.7 kJ mol^−1^. Contacts to molecules I and J involve longer C4H4∙∙∙I interactions at 3.59 Å and have energies of −7.6 kJ mol^−1^. The longest contacts are formed in offset π∙∙∙π stacking interactions, at 4.040(2) Å in molecules K and L, with total molecular interaction energies of −6.8 kJ mol^−1^. 

**Figure 4:**
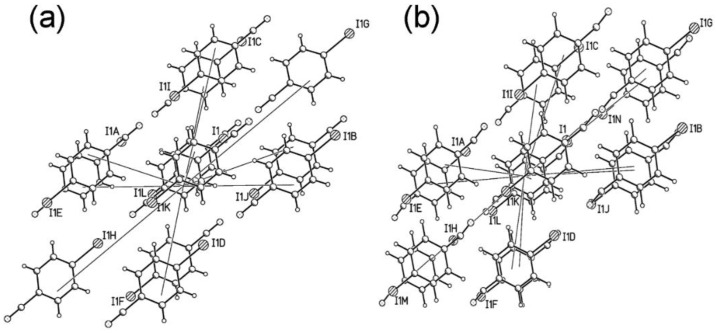
First coordination spheres of (**a**) Phase I at 5.0 GPa. Notice the ABC layering sequence in the vertical direction. (**b**) Phase II at 5.5 GPa.

Overall the first coordination sphere contains 12 molecules broken down into four pairs and one quartet of symmetry related interactions to give five unique dimer pairs. Each molecule is surrounded by six others forming a layer with three molecules above and three below the layer to give an approximately cubic close packed underlying topology (Figure 4).

### 2.2. Response of Phase I to Pressure

**Figure 5:**
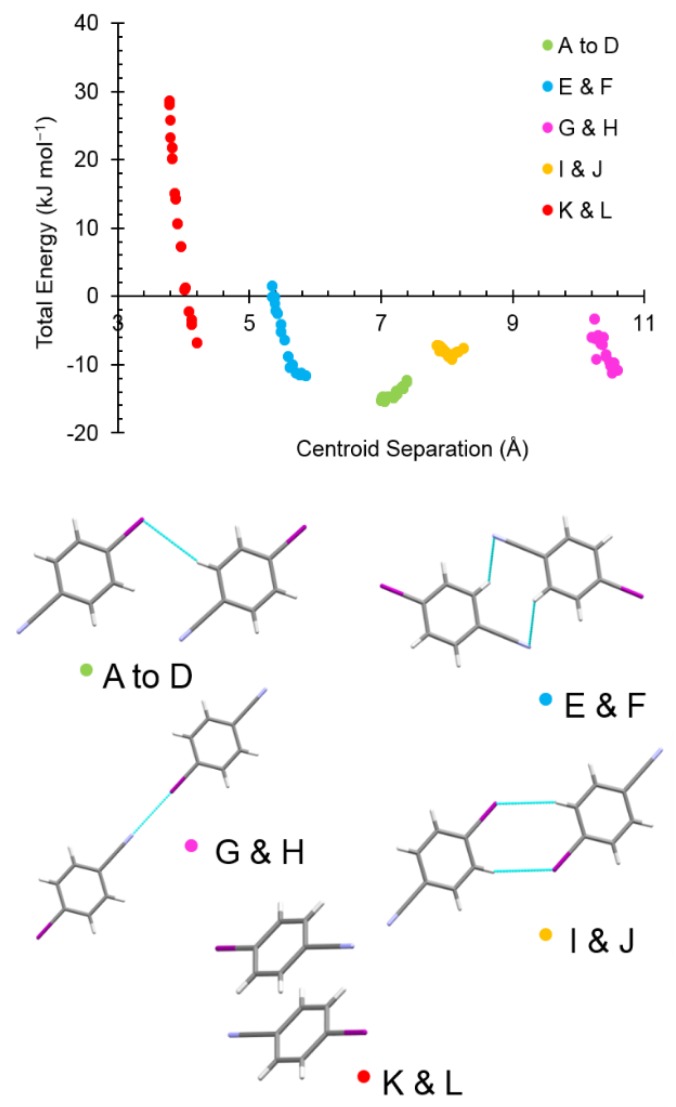
Graph of total interaction energy for the five most energetically significant dimers in 4-iodobenzonitrile phase I as a function of molecular centroid separation. The dimers shown are those of the ambient pressure structure.

The energies of the five unique dimers observed between ambient pressure and 5.0 GPa are plotted as a function of centroid separation in Figure 5, which also shows the structure of each dimer. Dimer energies at 5.0 GPa are listed in Table 2. There is a pronounced response to pressure in terms of total interaction energies in pairs E and F (Δ*E* = +10.2 kJ mol^−1^); G and H (Δ*E* = +7.5 kJ mol^−1^); and, K and L (Δ*E* = +34.9 kJ mol^−1^).

**Figure 6:**
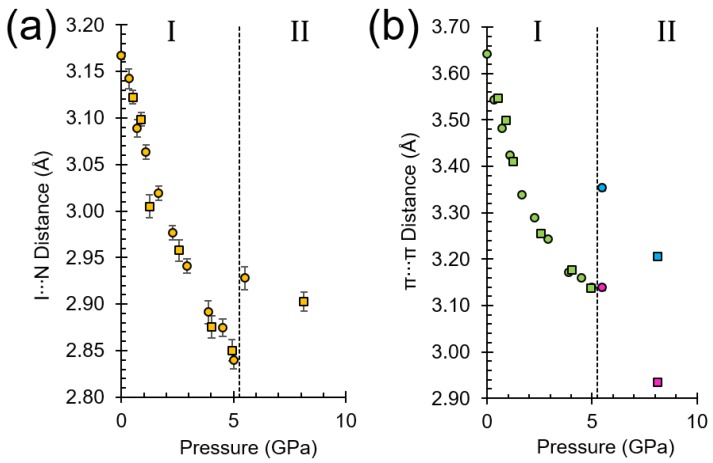
(**a**) Change in the axial I···N distance relating to contacts G and H as a function of pressure. Circles represent ex-situ data and squares represent in-situ data. The contacts remain related by symmetry in both phases. (**b**) The change in π···π stacking distances between the layers in contacts K and L as a function of pressure. Circles represent ex-situ collected data and squares in-situ collected data. K (blue) and L (purple) contacts split into two symmetry independent contacts in phase II.

The least sensitive of these involve the I∙∙∙N interactions to molecules G and H. The I∙∙∙N contact shortens from 3.168(4) to 2.840(1) Å between ambient pressure and 5 GPa (Figure 6a). The Coulombic energy is the largest term in these contacts and the overall molecular interaction remains slightly stabilising at 5.0 GPa with a total energy of −3.3 kJ mol^−1^. The frequency of the symmetric C-I/I∙∙∙N stretch increases with pressure, a reflection of the stiffening of intermolecular potentials as the molecules are pushed into closer proximity, reaching a value of 249 cm^−1^ at 4.3 GPa (the highest-pressure Raman measurement in phase I).

The contact which is most sensitive to pressure is with molecules K and L which interact via π∙∙∙π interactions which shorten from 3.6420(8) to 3.139(4) Å (Figure 6b). The overall interaction energy becomes quite destabilising at 5.0 GPa as the molecules are forced into a strongly repulsive regime (total interaction energy = +28.1 kJ mol^−1^). 

Of the remaining interactions, E and F which involve CH∙∙∙N interactions which shorten from 3.27 to 2.84 Å, are also quite sensitive to pressure. Dispersion is the largest energy term in these contacts, but the Coulombic contribution is also significant. Interaction energies in contacts A to D and I and J, are characterised by CH∙∙∙I interactions and hardly change with pressure at all. C4H4∙∙∙I7 interactions shorten from 3.29 to 2.88 Å and C3H3∙∙∙I7 interactions shorten from 3.59 to 3.09 Å to 5.0 GPa, and the total energies change by only −2.3 kJ mol^−1^ and +0.2 kJ mol^−1^, respectively. Molecules A to D, characterised by C4H4∙∙∙I7 interactions, are the only contacts seen to strengthen, albeit slightly.

The structural response to pressure thus favours compression of the weaker π∙∙∙π interactions over the I∙∙∙N halogen bond. The distinction is reflected in the changes in the unit cell dimensions.

### 2.3. Unit Cell Compression

**Figure 7:**
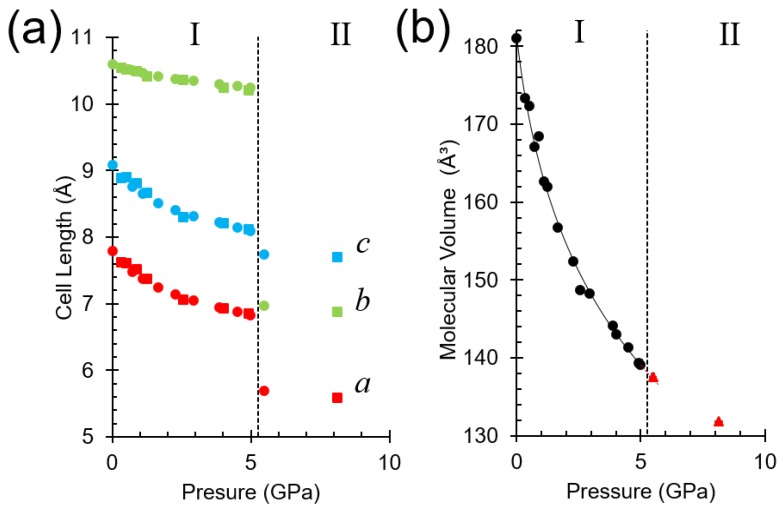
(**a**) Plot of unit cell parameters as a function of pressure. Ex situ crystal data are shown as circles and in situ crystal data are shown as squares. (**b**) Molecular volume of all phase I data, black circles, fitted to a 3rd order Birch-Murnaghan EOS and extrapolated to 5.7 GPa (red-dashed line). Phase II data are shown as red triangles. Error bars are obscured by data markers and the phase boundary is indicated by a dashed line in both figures.

The π∙∙∙π offset stacking interactions (molecules K and L), which show the largest decrease in distance with pressure, are expressed along the *a*− and *c*−axes, which compress by 12.3 and 10.9% up to 5.0 GPa. The least compressible interactions (I∙∙∙N) are generated by lattice translations along **b**, and this axis compresses by only 3.3% (Figure 7a). A fit of the variation of unit cell volume of phase I with pressure to a third order Birch-Murnaghan equation of state (EOS) is given in Figure 7b and yields a bulk modulus *K*_0_ = 6.5(6) GPa, with a pressure derivative *K*’ = 8.9(11). 

A small bulk modulus (<10 GPa) is typical of soft materials where dispersion forces dominate intermolecular interactions e.g., naphthalene and Ru_3_(CO)_12_ have values of 8.3(4) and 6.6 GPa, respectively [25,26]. Flexible intramolecular torsion angles can provide an additional mechanism for compression, as seen in the P and OP polymorphs of the prodigiously polymorphic compound ‘ROY’ (5-methyl-2-[(2-nitrophenyl)amino]-3-thiophenecarbonitrile), for which *K*_0_ = 6.0(7) and 4.3(3) GPa, respectively [27,28]. It assumes a higher value if additional intermolecular interactions such as H−bonding is present, e.g., the value for hydroquinone-formic acid clathrate is *K*_0_ = 13.6(4) GPa [29], and that of melamine is 12.0(5) [30]. Materials characterised by a mixture of dispersion and weaker H-bonds and have correspondingly lower bulk moduli, e.g., aniline (phase II) is 5.4(2) [31].

### 2.4. Formation of Phase II 

Phase I transforms to a new high-pressure polymorph (phase II) at 5.5 GPa, with sharp discontinuities in the unit cell dimensions (Figure 7a). Phase II is triclinic ($P\bar{1}$) with two molecules in the unit cell. The phase transition from phase I to II tended to lead to fragmentation of a single crystal of phase I. After some experimentation (see Section 4.2) a well-diffracting single crystal of phase II was obtained by in situ crystal growth in methanol-ethanol, followed by rapid increase in pressure to 8.1 GPa; the structure of phase II was solved from this data set. The solution was used to model the data obtained from a fragmented crystal at 5.5 GPa. 

**Figure 8:**
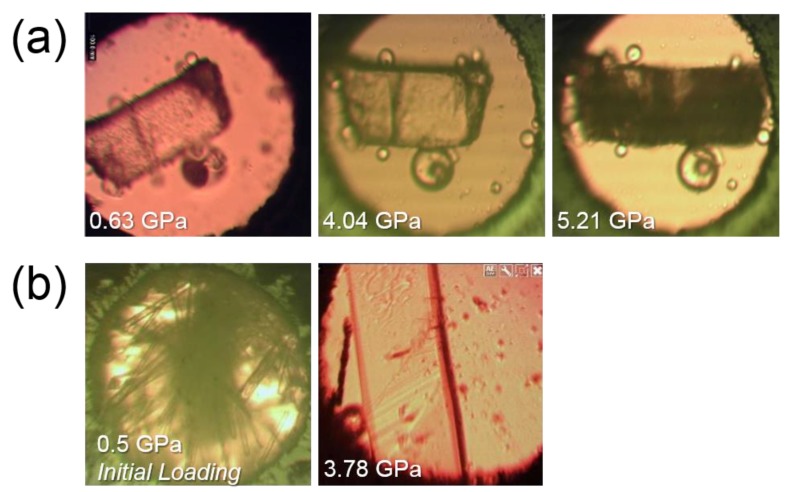
Crystals used in this study: (**a**) Ex situ crystal loaded in methanol-ethanol (Section 4.2i), the crystal transparency reduction is apparent at 5.21 GPa. (**b**) In situ crystal growth from melting the initial polycrystalline mass to 420 K and allowing to cool to room temperature repeatedly (Section 4.2iii).

**Figure 9:**
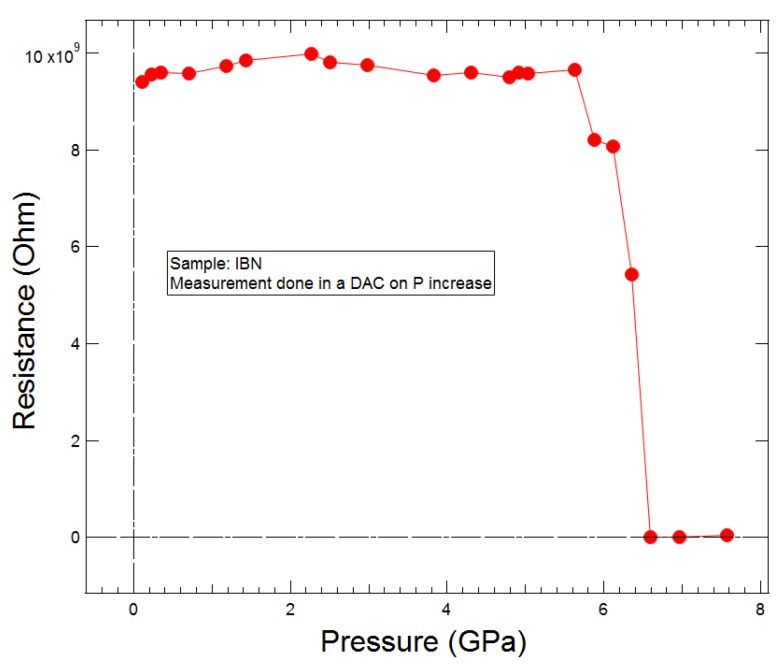
Resistance of polycrystalline 4-iodobenzonitrile as a function of pressure. A drop from 10^10^ to 8 × 10^9^ Ω occurs at the phase transition. The precipitous drop beyond 6 GPa comes from interference from the gasket.

**Figure 10:**
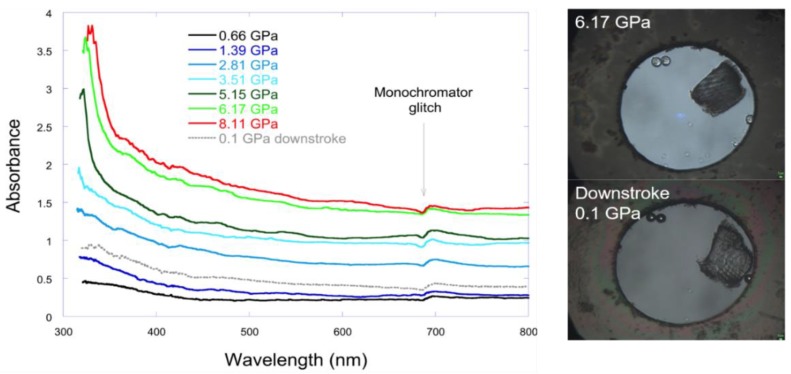
On the left: UV-Vis absorption spectra of 4-iodobenzonitrile as a function of pressure. On the right: images of the hydrostatic chamber with the sample and ruby spheres. The crystal does not completely recover its integrity after pressure release.

The phase transition causes the crystal to darken in colour (Figure 8a), accompanied by a marked decrease in resistance (Figure 9). However, the UV-visible spectra shown in Figure 10 indicate neither a significant redshift of the absorption edge nor any new absorbance features between 300 and 800 nm up to 8 GPa. The only changes occur in the background, which is related to the presence of light scattering by the sample. 

The creation of crystal domains, usual in reconstructive first order phase transitions involving anisotropic structures, gives rise to multiple scattering with the walls of the domains resulting in a clear darkening on the sample. Therefore, the sample darkening is not related to a change in band structure, but rather to formation of very small domains as the crystal passes through the phase transition. The change in microstructure reduces the transmittance of the sample by two orders of magnitude (Figure 10). 

**Figure 11:**
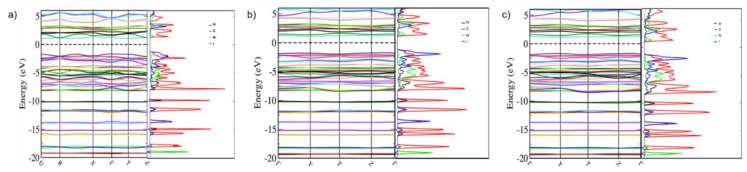
Band structure and electronic density of states of 4-iodobenzonitrile at (**a**) 5 GPa, (**b**) 5.5 GPa and (**c**) 8.14 GPa. The density of states are shown projected onto their atomic species where hydrogen—black, carbon—red, nitrogen—green and iodine—blue.

This conclusion is supported by the band structures of 4-iodobenzonitrile at 5, 5.5 and 8.1 GPa, shown in Figure 11. It is evident that each of the phases is an insulator, with calculated thermal bandgaps of 2.8 eV at 5 GPa, reducing to 2.5 eV at 8.14 GPa. These values will be underestimated compared to the true bandgap owing to the use of the PBE functional. Overall, the band structures show relatively flat bands, demonstrating that there is little dispersion in the crystal and that the bands can be viewed as molecular orbitals. The projected densities of states illustrate this, showing that each atomic species is responsible for the make-up of the bands. Interestingly, upon increasing pressure, the contribution of iodine to the HOMO band is markedly increased.

The clearest effect of the phase I → II transition on the Raman spectra (Figure 2) is an increase in intensity in the bands above 1500 cm^−1^ that correspond to a mixture of CC stretches and CCH bends in the phenyl group. The results of the DFT calculations indicate that a doubling of the intensity in this region would be expected, and the larger enhancement seen in the spectra in Figure 2 may additionally reflect the influence of sample orientation. The changes below 250 cm^−1^ are more dramatic, with the whole-molecule rocking region now extending beyond 200 cm^−1^ (Figure 3b). Three modes persist in the 250 cm^−1^ region, now all of the same symmetry (A_g_). The calculated frequency values, 248 cm^−1^ (intensity 131 Å^4^ amu^−1^), 254 (93) and 277 (33) agree within 10 cm^−1^ with the observed positions at 252, 263 and 282 cm^−1^. Animations of the modes indicate that the C-I/I···N stretch remains localised in the first, and strongest, of these bands. The other two modes are in- and out- of plane oscillations which cause bending at the I···N interaction.

High-pressure phases are occasionally recoverable at ambient pressure (some recent examples being glycolide, [32] mefanamic [33] and δ-*p*-aminobenzoic acids [34]). However, the Raman spectrum collected on complete pressure release resembles the ambient pressure single-crystal spectrum, indicating that the transition is reversible. The optical transmittance of the sample also recovers, but only partially (Figure 10). 

### 2.5. The Crystal Structure of Phase II

Pressures below 10 GPa can lead to significant changes in intramolecular bonding interactions organic materials, as was the case for example in syn-1,6:8,13-biscarbonyl [14] annulene [35]. In the present study control of the ratio of the numbers of data to refined parameters meant that it was necessary to apply restraints to the intramolecular bond distances and angles, and so it is not possible to determine if there are major changes in these parameters with pressure. This said, the nitrile group appears to be quite non-linear at 5.5 GPa with <C5−C6−N7 = 169(2)°. The non-linearity persisted when the structure of phase II was optimised by periodic DFT, with a magnitude which is smaller (4.4°) and more similar to the value 4(3)° obtained at 8.1 GPa. It is possible that the larger experimental value at 5.5 GPa is a consequence of the relatively low data quality obtained from the fragmented sample (*R*_1_ = 11.1%).

**Figure 12:**
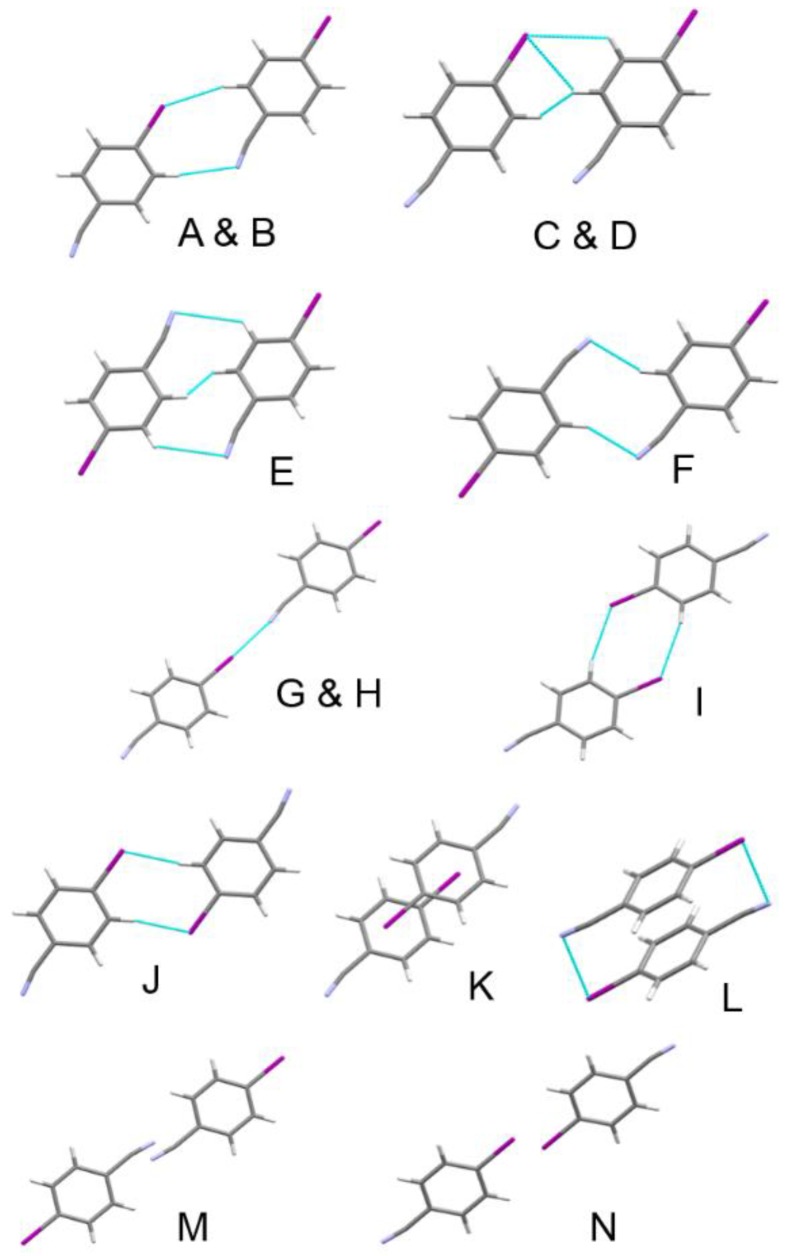
The most energetically significant dimers in the phase II structure of 4-iodobenzonitrile at 5.5 GPa.

Phase II exhibits the same four classes of short contact found in phase I (π···π, CH···I, CH···N and I···N) as well as some additional I···π and H···H contacts. The energetically most significant dimer interactions in are shown in Figure 12, with energies listed in Table 3. The contacts in Table 3 are labelled so that molecule A in phase II occupies a similar position in the first coordination sphere as molecule A in phase I etc. The contact labels in the two phases can thus be correlated directly (Figure 4). 

Like phase I, the structure of phase II consists of chains of molecules which interact via I∙∙∙N interactions (G and H). The chains are no longer strictly linear [<C2I1∙∙∙N7 = 166.2(5)°] and the I∙∙∙N distance increases over the course of the transition from 2.840(1) Å in phase I at 5.0 GPa to 2.928(10) Å in phase II at 5.5 GPa. Even at 8.1 GPa the distance is 2.90(2) Å (Figure 6a). The energy of the interaction does not change by much: −3.3 kJ mol^−1^ in phase I at 5.0 GPa and −5.6 kJ mol^−1^ in phase II at 5.5 GPa. These figures are lower than in a range of ambient-pressure structures with differing sigma−hole strengths in which the I∙∙∙N distances varied between 2.95 and 3.15 Å and the energies from −30.2 to −15.1 kJ mol^−1^ [15]. Consistent with the modest strengthening, the frequency of the C-I/I···N stretching mode is similar but slightly higher than at 4.3 GPa (252 versus 249 cm^−1^).

Each chain is surrounded by six other chains. The four equivalent inter-chain interactions of phase I labelled A-D and mediated by short I∙∙∙H and N∙∙∙H contacts split in phase II into two pairs of contacts. The A/B pair is similar to phase I, but C and D become off-set stacking interactions with a shorter centroid-centroid distance and a slightly destabilising energy. The pair of antiparallel off-set stacking interactions E/F in phase I split into two inequivalent contacts in phase II. Contact E is similar to that in phase I, but interaction F becomes longer and more coplanar. The change in contact F is partly responsible for driving the phase transition (see below).

The degenerate pair of contacts I and J in phase I split into symmetry-inequivalent interactions in phase II, but their character and energies are largely unaffected. The pair of antiparallel stacking contacts K/L in phase I, which become strongly destabilising in phase I at 5.0 GPa, split into two stacking interactions (Figure 6b). In contact K the C–I bond of one molecule moves above the phenyl ring of the other, with the slippage between the rings changing from 1.747 to 3.056 Å. The stacking distance increases from 3.139(4) to 3.353(6) Å, and the energy becomes less positive. In L the slippage is slightly smaller (1.614 Å) with an unchanged stacking distance of 3.139(6) Å and the interaction energy becomes more positive. 

Two new long contacts where the shortest distances are formed between pairs of iodine and nitrogen atoms also emerge as two additional molecules (M and N) enter the first coordination sphere. The interactions are long [N7∙∙∙N7 = 4.10(4) Å in M and I1∙∙∙I1 = 4.241(7) Å in N] and the energies are small but stabilising. The first coordination sphere in phase II thus contains fourteen molecules. It is like that of Phase I, with a central six molecule layer but now with four molecules above and four molecules below the central layer instead of three, resulting in a change of the underlying topology from pseudo cubic close packed in phase I to body centred cubic in phase II (Figure 4b).

### 2.6. The Driving Force of the Phase I-to-II Transition

The orientation matrices for the diffraction patterns obtained before and after the transition yield the following relationship between the basis vectors of phases I and II:

$$\left(\begin{array}{ccc} -0.478 & 0.403 & -0.423\\ \;\;\;0.470 & 0.421 & \;\;\;0.917\\ \;\;\;0.512 & 0.499 & -0.189 \end{array}\right)$$

The patterns were measured at different pressures (5.0 and 5.5 GPa), but the elements of this matrix are still rather far from rational fractions, meaning that there is not a simple symmetrical relationship between the phases. This is further evidence that the phase transition is first order and reconstructive, supporting the conclusions made in Section 2.4. 

The extrapolated molecular volume of phase I at 5.5 GPa is 137.3(3) Å^3^. The observed value of the molecular volume at 5.5 GPa is the same, within error, at 137.6(3) Å^3^. The negligible change in molecular volume at the phase transition is significant because it indicates that, unlike most high-pressure phase transitions, the dominating thermodynamic factor is not volume minimisation. The volume in phase II at 8.1 GPa appears to lie on the same trend-line as those in phase I, but with only two data points available it is not possible to determine an equation of state for phase II.

In order to identify the likely ‘driving force’ of the phase transition the intermolecular interaction energies in phase I were calculated using a structure in which the unit cell dimensions of phase I were extrapolated to 5.5 GPa using third-order Birch-Murnaghan equations of state (EoSFIT) [36]. The estimated cell dimensions were *a* = 6.7923 Å, *b* = 10.2202 Å, *c* = 8.0533 Å and β = 100.7°. The structure was optimised by periodic DFT, and intermolecular energies calculated using PIXEL. Intermolecular energies were likewise calculated following optimisation of the experimentally-determined phase II structure at the same pressure. The lattice energy of phase I was +4.1 kJ mol^−1^ and that of phase II −0.9 kJ mol^−1^, reproducing the expected energetic ordering of phases I and II at the phase transition. The difference in DFT energies was −0.2 kJ mol^−1^.

A comparison of contact energies within the first coordination sphere (Table 4, a more detailed listing is available in the ESI, Table S3) shows that overall the contacts in phase II are more stabilising by 12.9 kJ mol^−1^. The largest stabilising changes over the course of the I to II transition occur for the contacts F and K. In both cases the stabilising components of the molecule-molecule energies become less negative, but at the same time the repulsion terms also become less positive (a full breakdown given in Table S4 in the ESI). Both effects can be traced to the larger centroid-centroid separations for the contacts in phase II, contact F changing from 5.38 to 6.61 Å and K from 3.73 to 4.81 Å. 

Both contacts F and K are destabilising in phase I at 5.5 GPa. After the transition F becomes stabilising, while K remains destabilising but less so than in phase I. The transition can thus be described as being driven by relief of interactions that had become destabilising on compression of phase I.

## 3. Conclusions

Taylor has compared the frequency of occurrence of intermolecular contacts in crystal structures to the frequency expected if determined solely by the exposed surface areas of atoms [37]. Highly preferred interactions occur much more often than would be expected on the basis of random packing arrangements, an effect which can be quantified with a metric *R*_F_ which adopts a value of > 1 if interactions are formed more frequently than would be expected by chance. Hydrogen bonds have the highest values of *R*_F_, occupying 9 out of the top 10 highest-ranked interactions, for example H-bonds to Br^−^, oxygen and sulfur are 10.9, 5.8 and 2.9, respectively. The I∙∙∙N halogen bond, for which *R*_F_ = 5.4, is the only non-hydrogen bond to occur in Taylor’s Top Ten, with a similar frequency to H-bonds involving oxygen and nitrogen.

4-Iodobenzonitrile is ideal for studying the mechanical properties of this, the most consistently-formed, halogen bond because it forms crystals in which I∙∙∙N bonds are exactly aligned with the *b*-axis of its monoclinic unit cell. Symmetry, in the form of Neumann’s Principle, demands that this direction must be one of the principal directions of anisotropic strain. Therefore comparing compression along **b** with that along **a** and **c** enables the response to pressure of the I∙∙∙N halogen bond to be compared directly to that of interactions mediated by other common classes of interaction such as π-stacking and CH∙∙∙N and CH∙∙∙I contacts. The gradients of the lines in the ‘phase I’ panel of Figure 7a graphically illustrate the low compressibility of the I∙∙∙N bonds compared to the other contacts in the crystal structure.

Even though the I∙∙∙N interaction is the least compressible interaction, that does not mean that it is the strongest. PIXEL calculations show that molecule-molecule energy within the halogen-bonded chains is essentially the same as in the more compressible contacts (Table 2). The response of different contacts to pressure measures their deformability rather than their strength (i.e. energy), characterising the shape of an intermolecular potential rather than its depth. Deep wells are not necessarily steep wells. 

PIXEL calculations show that the Coulombic contribution to the I∙∙∙N interaction is twice that of the dispersion term, and that this weighting increases with pressure. Dispersion is the largest term in the other contacts, but here too, the contribution of electrostatics increases with pressure. Interactions which are dominated by dispersion are often found to be deformable because dispersion imposes no directional restrictions, whereas there is usually a distinct orientational preference in electrostatic interactions, the linear geometry of the halogen bonding studied here, for example, being dictated by the spatial characteristics of the lone pair on N and σ-hole on I. However, the halogen bond remains linear up to 5 GPa and its incompressibility may simply reflect that at ambient pressure the I∙∙∙N distance is already 0.36 Å within the sum of the van der Waals radii of I and N, a figure which rises to 0.69 Å at 5 GPa. There is thus less ′room for manoeuvre′ than in the other interactions for which the minimum figure is 0.30 Å even at 5 GPa. 

The I∙∙∙N interaction remains stabilising (just) throughout the pressure range of this study. The same is not true of the N∙∙∙H and π-stacking interactions, which have become destabilising at 5 GPa. On increase of pressure to 5.5 GPa the monoclinic phase 4-iodobenzonitrile undergoes a phase transition to a triclinic phase. The I∙∙∙N-linked chains become less linear, but the PIXEL calculations show that the transition is driven by relief of the destabilising N∙∙∙H and π-stacking interactions. The reduction in molecular volume which must occur at the transition is within the precision of our measurements, the apparent small increase relative to the extrapolated phase I volume is just statistical noise. Unlike most other high-pressure phase transitions (e.g., in mephodrone hydrogen sulfate [38] or L-serine [39,40]) the one in 4-iodobenzonitrile thus appears to be driven by the contribution of the intermolecular interactions to the free energy rather than the *P*Δ*V* term; a similar feature was observed in a phase transition in salicylaldoxime [41].

## 4. Experimental

### 4.1. High-Pressure Experiments 

Single crystal X-ray diffraction, Raman and UV-Vis absorption spectroscopy at high pressure were performed in Merrill-Bassett or Boehler-Almax type diamond anvil cells (DACs) equipped with 0.6 mm culet type Ia Boehler-Almax cut diamonds. Tungsten gaskets with holes of diameter 0.25–0.35 mm and thickness 0.1–0.12 mm formed the sample chambers and the hydrostatic pressure transmitting media, were either 1:1 *n*-pentane:isopentane, 4:1 methanol-ethanol or spectroscopic paraffin oil. Pressures were determined by the ruby fluorescence method [42].

### 4.2. Recrystallizations and Compression Conditions

Over the course of this work the response of 4-iodobenzonitrile to high pressure was studied under four different sets of conditions. 

(*i*) *Ex situ crystal growth followed by compression in pentane-isopentane:* 4-Iodobenzonitrile (97%, Sigma-Aldrich, Irvine, UK) was first recrystallized by solvent diffusion of *n*-pentane in dichloromethane solution to give colourless, rectangular-prismatic crystals of the ambient-pressure phase (phase I). Initial ambient and high-pressure X-ray diffraction data were collected on a crystal of dimensions 0.1 × 0.2 × 0.2 mm^3^ in *n*-pentane:iso-pentane to 5.0 GPa using a lab source diffractometer (see below). Beyond 5.0 GPa the sample darkened in colour and diffraction quality decreased dramatically (Figure 8a) but there was nevertheless some evidence of crystallinity in the form of weak diffraction at low angle. A second series of measurements was therefore carried out using methanol-ethanol as a hydrostatic medium with the aim of obtaining better quality data above 5 GPa [43].

(*ii*) *Ex situ crystal growth and compression in methanol-ethanol:* 4-Iodobenzonitrile was recrystallized by slow evaporation of a 4:1 methanol-ethanol solution to give colourless, rectangular prism shaped crystals of phase I. Ambient and high-pressure X-ray diffraction data were collected using a crystal (0.1 × 0.1 × 0.1 mm^3^) and the mother liquor from the crystal growth as the pressure- transmitting medium. Data were collected to 5.5 GPa using synchrotron radiation (see below). A new phase (phase II) formed at 5.5 GPa but the sample contained numerous quite weakly-scattering domains. We therefore turned to in situ crystal growth with the aim of obtaining an improved data set. 

(*iii*) *In situ crystal growth and slow compression in methanol-ethanol:* A 4-iodobenzonitrile in 4:1 methanol-ethanol solution was loaded in a DAC to give a polycrystalline mass on compression at 0.5 GPa. A colourless crystal measuring 0.12 × 0.18 × 0.22 mm^3^ was obtained by repeatedly heating the DAC to 420 K and cooling to room temperature (Figure 8b). High-pressure X-ray diffraction data were collected to 4.9 GPa in ca. 0.9 GPa steps using synchrotron radiation. The crystal broke apart above 5 GPa and no discernible diffraction data could be obtained. 

(*iv*) *In situ crystal growth followed by rapid compression in methanol-ethanol:* A small single crystal (~0.05 mm^3^) was regrown in situ but rapidly compressed to 8.1 GPa from ambient pressure in one step. High-pressure diffraction measurements were performed immediately on a lab source diffractometer to give sufficiently good data for structure solution and refinement of phase II. 

### 4.3. Single Crystal X-ray Diffraction

Single crystal diffraction data were collected using both sealed-tube and synchrotron X-radiation. The synchrotron data were collected on Beamline 11.3.1 at the Advanced Light Source on a D8 diffractometer (Bruker, Madison, WI, USA) with silicon (111) monochromated synchrotron radiation, wavelength 0.7749 Å (energy = 16.5 keV) and PHOTON-II detector. Shutterless ω-scans with different 2θ and ϕ-offsets were performed at step widths of 0.3° with exposure times of 1 s. The sealed-tube data sets were collected using a Bruker APEX-II diffractometer with graphite monochromated Mo-Kα radiation (λ = 0.71073 Å) and exposure times of 30 s. 

Diffraction images were integrated using SAINT with dynamic masks generated by ECLIPSE to mask shaded detector areas [44,45]. The multi-scan procedure SADABS [46] was used to treat cell and sample absorption errors. The structure of phase I was solved using dual-space methods (SHELXT) [47] and that of phase II at 8.1 GPa by simulated annealing (DASH) [48]. Structures were refined by full-matrix least-squares on |*F*|^2^ (SHELXL) [49] using the ShelXLe graphical user interface [50].

Non-hydrogen intramolecular bond distances in each high-pressure model were restrained to those of the ambient temperature-pressure structure. In phase I the molecule has. 2. site symmetry; in phase II it occupies a general position but was restrained to have *C*_2v_ symmetry during refinement. Only the iodine atom was refined with anisotropic displacement parameters, with the light-atoms restrained to have similar isotropic displacement parameters in the high-pressure structure refinements; the ambient temperature-pressure structure was refined with anisotropic displacement parameters for non-hydrogen atoms. All H-atoms were placed in calculated positions and allowed to ride on their parent atoms. The phase I to II transition is reconstructive and there is no rational geometric relationship between the lattices (see Section 2.6); it was found unnecessary to model twinning in phase II. 

Crystal and refinement data immediately below and above the phase transition are collected in Table 5, a full set of parameters for the 18 data sets collected over the course this study is available in the electronic supplementary information (ESI). CCDC 1911442-1911459 also contains the supplementary crystallographic data for this paper. These data can be obtained free of charge online [51].

### 4.4. Raman and UV-Vis Spectroscopy

High-pressure Raman spectra were collected in parallel to the single-crystal diffraction experiments during the study using the crystal grown ex situ in methanol-ethanol as a pressure transmitting medium [method (ii) above]. The instrument used was a LabRAM HR Evolution Raman spectrometer (Horiba, Longjumeau, France) using an 1880 lines mm^−1^ grating and 633 nm excitation. The range was 50 to 3400 cm^−1^_._ A BXFM-ILHS microscope (Olympus, Waltham, MA, USA) with a 50× long working distance objective was used for laser focusing to a spot size of *ca.* 2 μm on to the sample. Spectra were collected on compression up to 9.7 GPa and on decompression to ambient pressure at 298 K.

UV-Vis absorption spectra at high pressure and room temperature were recorded using a custom-made apparatus. The modulated light from deuterium and tungsten lamps was dispersed with a monochromator and focused on the sample with a reflective objective. The transmitted light was collected with another reflective objective and the signal detected with a photomultiplier was synchronously detected with a lock-in amplifier. The experiments were carried out using paraffin oil (Merck, Darmstadt, Germany) as transmitting medium. The pressure and hydrostatic conditions were checked through the shift and bandwidth of ruby R-lines luminescence, respectively.

### 4.5. High-Pressure Conductivity Measurements 

**Figure 13:**
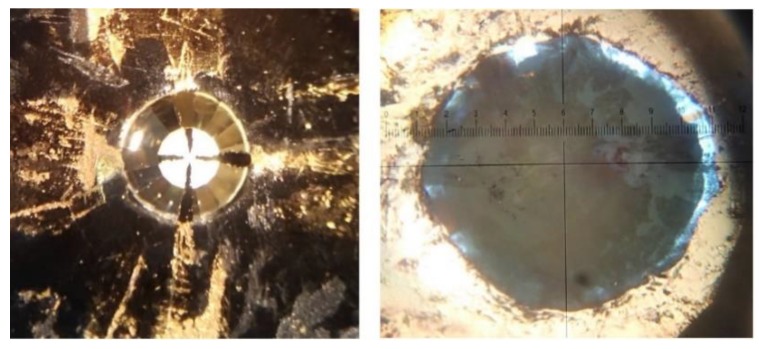
On the left: Diamond with gold sputtered contacts (no sample). On the right: IBN sample within a gasket hole at 5 GPa. The PVC layer is seen in the transparent light blue areas between the inner gasket hole and sample.

Electrical resistance was measured on a compressed powder sample using a four-probe method in a Merrill-Bassett DAC with 800 μm culet anvils (Figure 13). Gold contacts were made on one of the anvils through a custom-made mask using a sputter coater. Daphne 7373 oil was used as a pressure medium. A ruby chip was loaded into the sample space and pressure was measured using ruby fluorescence. The metallic parts of the cell and gasket were electrically insulated to avoid short circuits. The electrical resistance of the sample was measured using a 6517A electrometer (Keithley, Cleveland, OH, USA) using the constant voltage method. The electrical resistance was measured with increasing pressure. At just above 5 GPa we observed a sudden drop in resistance which is also accompanied by the darkening of the sample.

### 4.6. PIXEL Energy Calculations

Molecular electron densities were calculated by quantum chemical methods at each pressure point by GAUSSIAN09 [52] with the MP2/DGDZVP level of theory and basis set. CH distances were reset to 1.083 Å for structures determined experimentally with X-ray diffraction, but left unchanged for structures which had been optimised by periodic DFT. The electron density grid obtained from Gaussian was in steps of 0.06 Å and condensation level of 4 was used for the PIXEL calculations (CLP-PIXEL) [53,54]. The cluster radius was 15 Å. A breakdown of the interaction energies within the first coordination spheres of phase I at ambient pressure and 5.0 GPa and phase II at 5.5 GPa are given in Table 1, Table 2 and Table 3. 

PIXEL has been used previously to study interactions involving iodine [15,55], and the ability of the method to reproduce experimental sublimation energies of iodine compounds is assessed in the ESI. For 4-iodobenzonitrile itself the lattice energy calculated by PIXEL is −62.5 kJ mol^−1^, which is smaller in magnitude than the experimental enthalpy of sublimation, 88.0(3) kJ mol^−1^, determined using Knudsen effusion methods by Rocha et al. [56]. However, the difference is consistent with the performance of PIXEL for other iodine containing compounds. Rounding errors in the PIXEL energy calculations also occur in the presence of iodine because of its large size and high polarizability and results in small differences in the calculated energies of symmetry equivalent molecules, for example, symmetry related contacts E and F at 0.0 GPa (Table 1) have Coulombic energy terms of −7.7 and −7.5 kJ mol^−1^. In these cases, the calculated PIXEL energies are averaged to give −7.6 kJ mol^−1^.

### 4.7. Periodic Density Functional Theory (DFT) Calculations

Geometry optimisations, vibrational frequency and Raman intensity calculations were carried-out using the plane-wave pseudopotential method in the CASTEP code [57] as incorporated in Materials Studio version 2017 [58]. The PBE exchange-correlation functional was used [59] with norm-conserving pseudopotentials and a basis set cut-off energy of 1020 eV. Brillouin zone integrations were performed with a Monkhorst-Pack **k**-point grid spacing of 0.07 Å^−1^ [60]. The starting coordinates were taken from the single-crystal diffraction structures at 0.0 and 5.5 GPa and optimised using the Tkatchenko-Scheffler correction for dispersion (DFT-D) [61]. The cell dimensions were fixed to the experimental values, and the space group symmetry was retained. For geometry optimisations the convergence criteria were 5 × 10^−6^ eV/atom in energy, 0.01 eV Å^−1^ for force, 5 × 10^−4^ Å for displacement and 5 × 10^−7^ eV/atom for self-consistent field convergence. Prior to calculation of the Raman spectra, these were tightened to 1 × 10^−8^ eV/atom, 0.003 eV Å^−1^ and 1 × 10^−10^ eV/atom, respectively [62]. Frequencies were calculated at the Γ-point only for comparison with the experimental Raman spectra.

### 4.8. Band Structure Calculations

Band structure and projected density of state (pdos) calculations were performed using CASTEP (version 16.11), with the PBE exchange-correlation functional. Norm-conserving potentials built into CASTEP and a plane wave basis set energy cut-off of 1300 eV were used. For the band structures, **k**-points were sampled along high symmetry paths (U-R-X-Γ-Y-Z) and (Γ-Y-T-Z-Γ) for the phases I and II, respectively. Projected densities of states were generated from a subsequent band-structure calculation using OptaDOS [63,64]. 

### 4.9. Other Programs Used

OLEX2 [65] and MERCURY [66] were used for data visualisation, and sample geometries analysed using PLATON [67]. Equation-of-state calculations were performed by EOSFIT [36]. MOGUL [68], and CONQUEST [69] were used to survey the Cambridge Structural Database (CSD) [70].

## Figures and Tables

**Table 1:** PIXEL calculated dimer interaction energies of the phase I polymorph at 0.0 GPa. All energies are in kJ mol^−1^.

Label	Centroid Distance (Å)	Symmetry	Coulombic	Polarization	Dispersion	Repulsion	Total	Contacts
A	7.398	−1/2+x,1/2+y,−1/2+z	−8.4	−3.2	−10.6	9.6	−12.6	I1∙∙∙H4C4 = 3.29 Å
B		1/2+x,−1/2+y,1/2+z						
C		−1/2+x,−1/2+y,−1/2+z						
D		1/2+x,1/2+y,1/2+z						
E	5.864	1/2−x,3/2−y,1/2−z	−7.6	−3.1	−12.6	11.6	−11.7	2 × long N7∙∙∙H4C4 =
F		1/2−x,3/2−y,3/2−z						2 × 3.27 Å
G	10.592	x,−1+y,z	−19.0	−6.6	−8.8	23.6	−10.8	I1∙∙∙N7 = 3.168(4) Å
H		x,1+y,z						
I	8.248	1/2−x,1/2−y,1/2−z	−5.4	−2.0	−12.5	12.3	−7.6	2 × long I1∙∙∙H3C3 =
J		1/2−x,1/2−y,3/2−z						2 × 3.59 Å
K	4.205	1−x,1−y,1−z	−15.7	−5.8	−30.9	45.6	−6.8	Cg∙∙∙Cg = 4.040(2) Å
L		−x,1−y,1−z						

**Table 2:** PIXEL calculated dimer interaction energies of the phase I polymorph at 5.0 GPa, immediately below the phase transition. All energies are in kJ mol^−1^.

Label	Centroid Distance (Å)	Symmetry	Coulombic	Polarization	Dispersion	Repulsion	Total	Contacts
A	7.009	−1/2+x,1/2+y, −1/2+z	−19.5	−10.7	−20.3	35.6	−14.9	I1∙∙∙H4 = 2.88 Å &
B		1/2+x, −1/2+y,1/2+z						N7∙∙∙H3 = 2.83 Å
C		−1/2+x, −1/2+y, −1/2+z						
D		1/2+x,1/2+y,1/2+z						
E	5.349	1/2−x,3/2−y,1/2−z	−28.0	−14.6	−29.6	73.7	1.5	2 × long N7∙∙∙H3C3 = 2 × 2.90 Å
F		1/2−x,3/2−y,3/2−z						2 × N7∙∙∙H4C4 = 2 × 2.84 Å
G	10.239	x, −1+y,z	−43.7	−17.5	−15.3	73.2	−3.3	I1∙∙∙N7 = 2.840(1) Å
H		x,1+y,z						
I	7.862	1/2−x,1/2−y,1/2−z	−24.0	−10.4	−27.4	54.4	−7.4	2 × I1∙∙∙H3C3 = 2 × 3.09 Å
J		1/2−x, 1/2−y, 3/2−z						
K	3.781	1−x,1−y,1−z	−78.7	−28.4	−65.2	200.4	28.1	Cg∙∙∙Cg = 3.593(6) Å
L		−x,1−y 1−z						

**Table 3:** PIXEL calculated dimer energies of the phase II polymorph at 5.5 GPa, immediately above the phase transition. All energies are in kJ mol^−1^.

Label	Centroid Distance (Å)	Symmetry	Coulombic	Polarization	Dispersion	Repulsion	Total	Contacts
A	7.728	x,y,1+z	−17.8	−8.9	−18.3	32.6	−12.5	I1∙∙∙H4 = 2.94 Å &
B		x,y,−1+z						N7∙∙∙H9 = 2.77 Å
C	5.692	−1+x,y,z	−37.5	−17.4	−40.7	98.6	3.0	I1∙∙∙H8C8/H9C9 = 2.98/3.07 Å &
D		1+x,y,z						H3∙∙∙H8 = 2.38 Å
E	5.242	2−x, 1−y,1−z	−25.4	−15.1	−31.2	71.6	−0.1	2 × N7∙∙∙H3 = 2 × 2.88 Å &
								H4∙∙∙H8 = 2 × 2.44 Å
F	6.474	3−x,−y,1−z	−24.3	−9.4	−18.5	34.0	−18.2	2 × N7∙∙∙H8 = 2 × 2.50 Å
G	10.292	−1+x,y,−1+z	−33.9	−13.7	−13.2	55.3	−5.6	I1∙∙∙N7 = 2.928(10) Å
H		1+x,y,1+z						
I	8.287	1−x,1−y,−z	−14.9	−8.6	−23.8	41.1	−6.3	2 × I1∙∙∙H3 = 2 × 3.09 Å
J	7.240	2−x,−y,−z	−29.8	−17	−31.1	72.6	−5.2	2 × I1∙∙∙H9 = 2 x2.87 Å
K	4.894	2−x,1−y,−z	−70.5	−23.6	−56.8	172.5	21.6	2 × I1∙∙∙Cg = 3.38(2)
L	3.337	2−x,−y,1−z	−59.6	−24.2	−57.7	188.3	46.7	Cg∙∙∙Cg = 3.530(8) Å &
								I1∙∙∙N7 = 3.33(2) Å
M	7.832	3−x,1−y,1−z	−1.8	−0.7	−2.6	0.6	−4.4	long N7∙∙∙N7 = 4.10(4) Å
N	10.011	1−x,−y,−z	−4.6	−1.4	−7.3	11.8	−1.5	long I1∙∙∙I1 = 4.241(7) Å

**Table 4:** Comparison of energies within the first coordination spheres in phases I and II at 5.5 GPa. Energies are in kJ mol^−1^ and distances are in Å. Both structures were optimised by periodic DFT and the energies calculated using PIXEL.

Phase I at 5.5 GPa			Phase II at 5.5 GPa			Δ(II-I)
Contact	Distance	Total Energy	Contact	Distance	Total Energy	
A	6.984	−13.8	A	7.728	−12.2	1.6
B	6.984	−13.8	B	7.728	−12.2	1.6
C	6.984	−13.5	C	5.692	1.2	14.7
D	6.984	−13.5	D	5.692	1.2	14.7
E	5.381	3.4	E	5.296	−4.9	−8.3
F	5.382	2.5	F	6.606	−18.6	−21.1
G	10.22	−0.1	G	10.292	−6.3	−6.2
H	10.22	−3.7	H	10.292	−6.3	−2.6
I	7.774	−5.3	I	8.145	−6.1	−0.8
J	7.774	−5.3	J	7.158	−4.9	0.4
K	3.729	33.1	K	4.805	16.8	−16.3
L	3.729	35.3	L	3.375	50.4	15.1
			M	7.962	−3.7	−3.7
			N	9.878	−2.0	−2.0
Totals		5.3			−7.6	−12.9

**Table 5:** Experimental details for the ambient pressure structure, and structures immediately below and above the phase transition. For all structures: C_7_H_4_IN, M_r_ = 229.01. Experiments were carried out at 298 K. H-atom parameters were constrained. CCDC deposition numbers for the structures listed are 1911442, 1911457, and 1911458.

Pressure (GPa)	0.00	5.00	5.49
Phase	I	I	II
Crystal data			
Crystal system, space group	Monoclinic, *I*2/*a*	Monoclinic, *I*2/*a*	Triclinic, $P\bar{1}$
*a*, *b*, *c* (Å)	7.788 (2), 10.592 (3), 9.086 (3)	6.8316 (3), 10.2395 (6), 8.0931 (18)	5.692 (5), 6.9674 (11), 7.7283 (12)
α, β, γ (°)	90, 104.934 (9), 90	90, 100.696 (7), 90	65.412 (13), 80.98 (4), 87.77 (3)
*V* (Å^3^)	724.2 (4)	556.29 (13)	275.1 (3)
*Z*	4	4	2
Radiation type	Synchrotron, λ = 0.7749 Å	Mo *Kα*	Synchrotron, λ = 0.7749 Å
µ (mm^−1^)	5.39	5.63	7.09
Crystal size (mm)	0.20 × 0.02 × 0.02	0.20 × 0.10 × 0.10	0.1 × 0.1 × 0.1
Diffractometer	Bruker D8 with Photon II detector	Bruker *APEX* 2	Bruker D8 with Photon II detector
*T*_min_, *T*_max_	0.533, 0.746	0.601, 0.745	0.390, 0.745
No. of measured, independent and observed [*I* > 2σ(*I*)] reflections	3052, 741, 703	1028, 192, 189	549, 236, 197
*R*_int_	0.027	0.039	0.041
(sin θ/λ)_max_ (Å^−1^)	0.626	0.622	0.575
*R*_1_[*F*^2^ > 2σ(*F*^2^)], *wR*_2_(*F*^2^), *S*	0.016, 0.038, 1.13	0.043, 0.114, 1.19	0.111, 0.293, 1.34
No. of parameters	44	22	30
No. of restraints	0	13	34
Δρ_max_, Δρ_min_ (e Å^−3^)	0.22, −0.53	1.19, −0.56	1.87, −1.16

## Supplementary Materials

The following are available online, Figure S1: A comparison of the observed enthalpies of sublimation and calculated PIXEL lattice energies used in method validation, Table S1: A breakdown of the compounds and their observed enthalpies of sublimation used in PIXEL method validation, Figure S2: Comparison between PIXEL and DFT calculated total lattice enthalpies of the geometry optimised structures, Table S2: Lattice enthalpies of the geometry optimised structures as calculated by PIXEL and DFT, Table S3: Full breakdown of the comparison of energies within the first coordination spheres in phases I and II at 5.5 GPa, Table S4: Crystallographic experimental details.
