# Supplementary material for: The Effect of Pressure on Halogen Bonding in 4-Iodobenzonitrile
Source: Molecules. 2019 May 27;24(10):2018. doi: 10.3390/molecules24102018 (PMC6572472; doi:10.3390/molecules24102018)
Supplement: Supplementary file 1 [file molecules-24-02018-s001.pdf]

Supplementary Information

The Effect of Pressure on Halogen Bonding in 4-Iodobenzonitrile

Nico Giordano [1,2], Sergejs Afanasjevs [3], Christine M. Beavers [2,4,5,*], Claire L. Hobday [1], Konstantin V. Kamenev [3], Earl F. O'Bannon [2,4,6], Javier Ruiz-Fuertes [7], Simon J. Teat [2,*], Rafael Valiente [8] and Simon Parsons [1,*]

1 Centre for Science at Extreme Conditions and EaStCHEM School of Chemistry, The University of Edinburgh, King's Buildings, West Mains Road, Edinburgh, Scotland, EH9 3FD, UK; Nico.Giordano@ed.ac.uk (N.G.); Claire.Hobday@ed.ac.uk (C.L.H.)

2 Advanced Light Source, 1 Cyclotron Road, Lawrence Berkeley National Laboratory, Berkeley, CA 94720, USA; obannon2@llnl.gov

3 Centre for Science at Extreme Conditions and School of Engineering, The University of Edinburgh, King's Buildings, West Mains Road, Edinburgh, Scotland, EH9 3FD, UK; S.Afanasjevs@ed.ac.uk (S.A.); k.kamenev@ed.ac.uk (K.V.K.)

4 Department of Earth & Planetary Sciences, University of California, Santa Cruz, 1156 High Street Santa Cruz, CA 95064, USA

5 Present address: Diamond Light Source, STFC Rutherford Appleton Laboratory, Harwell Science and Innovation Campus, Harwell Oxford, Didcot, OX11 0QX, UK

6 Present address: Physical and Life Sciences, Physics Division, Lawrence Livermore National Laboratory, Livermore, CA 94551, USA

7 Dpto. DCITIMAC, Facultad de Ciencias, Universidad de Cantabria, 39005 Santander, Spain; javier.ruizfuertes@unican.es

8 Dpto. Física Aplicada, Facultad de Ciencias, Universidad de Cantabria-IDIVAL, 39005 Santander, Spain; rafael.valiente@unican.es

* Correspondence: christine.beavers@diamond.ac.uk (C.M.B.); sjteat@lbl.gov (S.J.T.); S.Parsons@ed.ac.uk (S.P.); Tel.: +44-(0)-131-650-5804 (S.P.)

Table of Contents

1. PIXEL Method Validation 2

Figure S1 A comparison of the observed enthalpies of sublimation and Calculated PIXEL energies used in method validation. 2

Table S1 A breakdown of the compounds and their observed enthalpies of sublimation used in PIXEL method validation. All energies given are in kJ mol^{-1} 3

2. Method Comparison: PIXEL and DFT Energies 4

Figure S2 Comparison between PIXEL and DFT calculated total lattice enthalpies of the geometry optimised structures 4

Table S2 Lattice enthalpies of the geometry optimised structures as calculated by PIXEL and DFT calculated lattice enthalpies 5

Table S3 Full Breakdown of the comparison of energies within the first coordination spheres in phases I and II at 5.5 GPa. 6

3. Crystallographic Tables 7

Table S4 Crystallographic Experimental Details. 7

4. References 12

1. PIXEL Method Validation

The literature was searched for iodine containing compounds in non-polar space groups for which enthalpies of sublimation had been determined experimentally, yielding nine compounds.[1] The observed energy value for each compound was taken as the mean of all published values and compared to its PIXEL calculated value. The iodine containing crystal structures were retrieved from the Cambridge Structural Database (CSD) [2] and electron densities calculated using the MP2/DGDZVP basis set by GAUSSIAN. Crystal lattice energies were then calculated using PIXEL.[3] A breakdown of the PIXEL calculated and observed energy values are plotted in Figure S1 with a goodness-of-fit of 0.68 which was deemed an acceptable level of agreement for iodine containing compounds. A breakdown of values is given in Table S1.

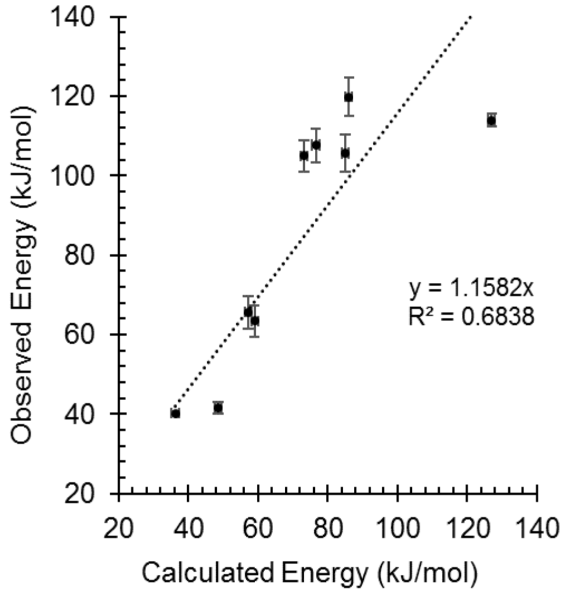

Figure S1 A comparison of the observed enthalpies of sublimation and Calculated PIXEL energies used in method validation.

Table S1 A breakdown of the compounds and their observed enthalpies of sublimation used in PIXEL method validation. All energies given are in kJ mol^{-1}.

Compound	CSD REFCODE	PIXEL Calculated	Observed Mean	Lowest Observed	Standard Error Obs. Values	No. of observations	Measurement Type
iodobenzene	REKYAI	48.5	41.55	40.00	1.55	2	ME, x
1,2-diiodoethane	ZZZFHE01	57.2	65.70	65.70	4.10	1	x
methyl iodide	MIMETH11	36.2	40.20	40.20	0.40	1	VG
2-iodobenzoic acid	OIBZAC01	73.2	104.95	92.60	4.03	4	ME, ME, C, DSC
5-iodocytosine	ZILBIF	127.0	114.00	114.00	1.50	1	x
3-iodobenzoic acid	ZZZOAE01	85.0	105.70	96.40	4.67	3	ME, ME, C
4-iodobenzoic acid	BENMOW07	76.5	107.70	99.30	4.25	3	ME, ME, C
5,7-diiodo-8-hydroxyquinoline	NEVMOR	86.0	119.87	110.90	4.70	3	ME, x, x
1,4-diiodobezene	ZZZPRO08	59.0	63.40	63.40	4.00	1	x

Measurement types are: mass effusion (ME), MKS baratron vacuum gauge (VG), differential scanning calorimeter (DSC) and calorimetric determination (C). 'x' denotes measurement types unavailable.

2. Method Comparison: PIXEL and DFT Energies

A second form of validation was obtained by comparing the changes in lattice energy calculated by PIXEL as a function of pressure with the total energies calculated by periodic DFT. Periodic DFT geometry optimisations were performed using CASTEP[4] on each of the experimentally determined structures. The unit cell dimensions were fixed at the experimental values, but the coordinates of the atoms were optimised. The parameters used for the calculations are given in Section 2.7 of the main paper. The DFT total energies were placed on the scale of the PIXEL calculations using:

$$U_{\text{DFT, scaled}} = U_{\text{DFT}} + \overline{U_{\text{PIX}} - U_{\text{DFT}}}$$

where $\overline{U_{\text{PIX}} - U_{\text{DFT}}}$ is the mean difference between the PIXEL and DFT enthalpies. The agreement between the scaled DFT and PIXEL energies is shown in Figure S2. The line of best fit between the two sets of energies is $0.88U_{\text{PIX}} - 4.32 = U_{\text{DFT, scaled}}$ and the values of R^2 is 0.9897. This level of agreement is similar to that seen for experimental sublimation energies.[5] Note that small changes in the internal geometry of the molecule, which will affect the total DFT energy, do not form part of the PIXEL calculations. Since such changes are within experimental error they have been neglected in this comparison.

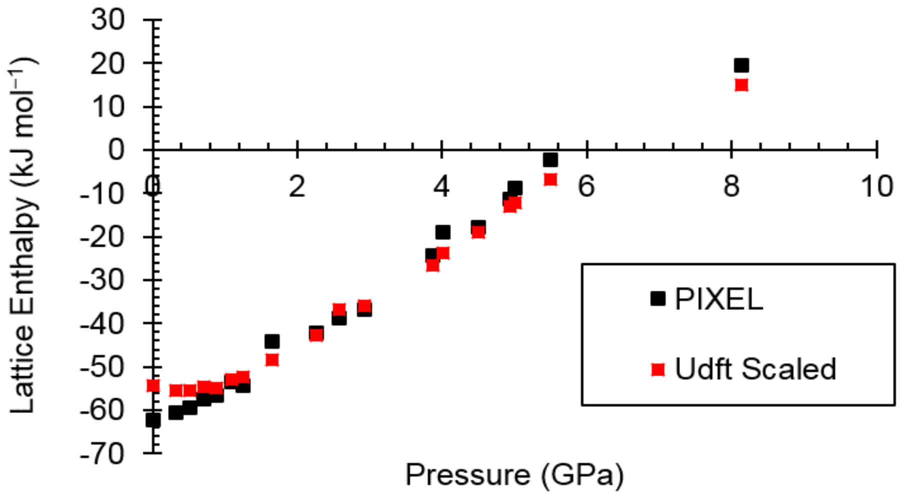

Figure S2 Comparison between PIXEL and DFT calculated total lattice enthalpies of the geometry optimised structures

Table S2. Lattice enthalpies of the geometry optimised structures as calculated by PIXEL and DFT calculated lattice enthalpies

Pressure (GPa)	Dispersion Corrected Final Energy (U_{DFT}, eV/cell)	Dispersion Corrected Final Energy (U_{DFT}, kJ/mol)	PIXEL Energy (U_{PIX}, kJ/mol)	$U_{PIX} – U_{DFT}$ (kJ/mol)	U_{DFT}, scaled (kJ/mol)
0.00	-3453.933782	-166626.4005	-62.2	166564.20	-54.36
0.33	-3453.958359	-166627.5861	-60.7	166566.89	-55.55
0.51	-3453.956278	-166627.4857	-59.5	166567.99	-55.45
0.71	-3453.942115	-166626.8025	-57.5	166569.30	-54.76
0.88	-3453.947561	-166627.0652	-56.7	166570.37	-55.03
1.09	-3453.901724	-166624.8539	-53.6	166571.25	-52.81
1.25	-3453.889456	-166624.2621	-54.4	166569.86	-52.22
1.66	-3453.807686	-166620.3173	-44.1	166576.22	-48.28
2.27	-3453.693496	-166614.8085	-42.2	166572.61	-42.77
2.57	-3453.568925	-166608.7989	-38.9	166569.90	-36.76
2.92	-3453.552524	-166608.0077	-36.7	166571.31	-35.97
3.87	-3453.360856	-166598.7611	-24.4	166574.36	-26.72
4.02	-3453.299873	-166595.8191	-18.9	166576.92	-23.78
4.51	-3453.201052	-166591.0517	-17.9	166573.15	-19.01
4.93	-3453.078869	-166585.1573	-11.2	166573.96	-13.12
5.00	-3453.058199	-166584.1602	-8.7	166575.46	-12.12
5.49	-3452.945897	-166578.7424	-2.2	166576.54	-6.70
8.14	-3452.493625	-166556.9237	19.5	166576.42	15.12
			Average $U_{PIX} – U_{DFT}$ =	166572.04	

Table S3. Full breakdown of the comparison of energies within the first coordination spheres in phases I and II at 5.5 GPa. Energies are in kJ mol^{-1} and distances are in Å. Both structures were optimised by periodic DFT and the energies calculated using PIXEL.

Phase I at 5.5 GPa							Phase II at 5.5 GPa							
Contact	Distance	Coul.	Pol.	Disp.	Rep.	Total	Contact	Distance	Coul.	Pol.	Disp.	Rep.	Total	Δ(II-I)
A	6.984	-21.6	-11.6	-21.2	40.6	-13.8	A	7.728	-18.8	-9.4	-19	35.0	-12.2	1.6
B	6.984	-21.6	-11.6	-21.2	40.6	-13.8	B	7.728	-18.8	-9.4	-19	35.0	-12.2	1.6
C	6.984	-21.1	-11.7	-21.2	40.5	-13.5	C	5.692	-36.6	-15.8	-38.3	91.8	1.2	14.7
D	6.984	-21.1	-11.7	-21.2	40.5	-13.5	D	5.692	-36.6	-15.8	-38.3	91.8	1.2	14.7
E	5.381	-27.7	-13.1	-27.9	72.1	3.4	E	5.296	-29.6	-13.8	-30.7	69.1	-4.9	-8.3
F	5.382	-27.8	-13.8	-28.1	72.3	2.5	F	6.606	-19.5	-7.1	-15.1	23.1	-18.6	-21.1
G	10.22	-45.1	-17.7	-15.4	78.1	-0.1	G	10.292	-32.1	-12.6	-12.5	50.9	-6.3	-6.2
H	10.22	-45.1	-17.7	-15.4	74.6	-3.7	H	10.292	-32.1	-12.6	-12.5	50.9	-6.3	-2.6
I	7.774	-26.7	-10.5	-27.8	59.6	-5.3	I	8.145	-19.3	-10.8	-25.8	49.7	-6.1	-0.8
J	7.774	-26.0	-11.3	-27.9	59.9	-5.3	J	7.158	-29.5	-15.6	-30.8	71.0	-4.9	0.4
K	3.729	-83.8	-32.3	-67.7	216.9	33.1	K	4.805	-63.4	-21.5	-55.1	156.8	16.8	-16.3
L	3.729	-84.1	-30	-67.4	216.9	35.3	L	3.375	-62.8	-24.8	-57.1	195.1	50.4	15.1
							M	7.962	-1.2	-0.8	-2.5	0.7	-3.7	-3.7
							N	9.878	-5.5	-1.7	-7.6	12.8	-2.0	-2.0
Totals		-451.7	-193	-362.4	1012.6	5.3			-405.8	-171.7	-364.3	933.7	-7.6	-12.9

The difference in energies of some symmetry equivalent contacts referred to in the experimental section has not been corrected.

3. Crystallographic Tables

Table S4. Crystallographic Experimental Details. For all structures: C_7H_4IN, Mr = 229.01. Experiments were carried out at 298 K. H-atom parameters were constrained.

Pressure (GPa)	0.00	0.33	0.51	0.71
Crystal data				
Crystal system, space group	Monoclinic, *I*2/*a*	Monoclinic, *I*2/*a*	Monoclinic, *I*2/*a*	Monoclinic, *I*2/*a*
a, *b*, *c* (Å)	7.788 (2), 10.592 (3), 9.086 (3)	7.6227 (9), 10.5434 (12), 8.892 (4)	7.6043 (6), 10.5114 (18), 8.8956 (15)	7.4794 (4), 10.5029 (5), 8.7497 (18)
α, β, γ (°)	90, 104.934 (9), 90	90, 104.016 (15), 90	90, 104.221 (5), 90	90, 103.496 (6), 90
V ($Å^3$)	724.2 (4)	693.4 (4)	689.25 (17)	668.36 (14)
Z	4	4	4	4
Radiation type	Synchrotron, λ = 0.7749 Å	Mo *K*α	Synchrotron, λ = 0.7749 Å	Mo *K*α
μ (mm^{-1})	5.39	4.52	5.66	4.69
Crystal size (mm)	0.20 × 0.02 × 0.02	0.20 × 0.10 × 0.10	0.22 × 0.18 × 0.01	0.20 × 0.10 × 0.10
Data collection				
Diffractometer	Bruker D8 with Photon II detector	Bruker *APEX* II	Bruker D8 with Photon II detector	Bruker *APEX* II
T_{min}, T_{max}	0.533, 0.746	0.626, 0.745	0.626, 0.745	0.531, 0.745
No. of measured, independent and observed [$I > 2\sigma(I)$] reflections	3052, 741, 703	819, 222, 204	1003, 357, 349	1259, 238, 223
R_{int}	0.027	0.027	0.021	0.030
$(\sin \theta/\lambda)_{max}$ ($Å^{-1}$)	0.626	0.617	0.626	0.623
Refinement				
$R[F^2 > 2\sigma(F^2)]$, $wR(F^2)$, S	0.016, 0.038, 1.13	0.029, 0.073, 1.19	0.025, 0.060, 1.17	0.035, 0.099, 1.27
No. of parameters	44	22	22	22
No. of restraints	0	13	13	13
Weighting scheme	$w = 1/[\sigma^2(F_o^2) + (0.0153P)^2 + 0.2329P]$ where $P = (F_o^2 + 2F_c^2)/3$	$w = 1/[\sigma^2(F_o^2) + (0.0298P)^2 + 5.9349P]$ where $P = (F_o^2 + 2F_c^2)/3$	$w = 1/[\sigma^2(F_o^2) + (0.0354P)^2 + 0.9045P]$ where $P = (F_o^2 + 2F_c^2)/3$	$w = 1/[\sigma^2(F_o^2) + (0.0525P)^2 + 5.0316P]$ where $P = (F_o^2 + 2F_c^2)/3$
$\Delta\rho_{max}$, $\Delta\rho_{min}$ (e $Å^{-3}$)	0.22, −0.53	0.30, −0.41	0.46, −0.50	0.49, −1.05

Table S4. *Cont.*

Pressure (GPa)	0.88	1.09	1.25	1.66
Crystal data				
Crystal system, space group	Monoclinic, *I*2/*a*	Monoclinic, *I*2/*a*	Monoclinic, *I*2/*a*	Monoclinic, *I*2/*a*
a, *b*, *c* (Å)	7.5174 (7), 10.487 (2), 8.8024 (18)	7.3792 (4), 10.4679 (6), 8.6488 (19)	7.3673 (11), 10.416 (4), 8.666 (3)	7.2480 (2), 10.4142 (3), 8.5115 (12)
α, β, γ (°)	90, 103.855 (5), 90	90, 103.128 (7), 90	90, 103.02 (1), 90	90, 102.576 (4), 90
V (Å^3)	673.7 (2)	650.61 (16)	647.9 (3)	627.05 (9)
Z	4	4	4	4
Radiation type	Synchrotron, λ = 0.7749 Å	Mo *K*α	Synchrotron, λ = 0.7749 Å	Mo *K*α
μ (mm^{-1})	5.79	4.82	6.02	5.00
Crystal size (mm)	0.22 × 0.18 × 0.01	0.20 × 0.10 × 0.10	0.22 × 0.18 × 0.12	0.20 × 0.10 × 0.10
Data collection				
Diffractometer	Bruker D8 with PHOTON II detector	Bruker *APEX* 2	Bruker D8 with Photon II detector	Bruker *APEX* 2
T_{min}, T_{max}	0.619, 0.745	0.541, 0.745	0.486, 0.745	0.617, 0.745
No. of measured, independent and observed [$I > 2\sigma(I)$] reflections	924, 343, 338	1172, 219, 206	530, 256, 252	1164, 218, 206
R_{int}	0.024	0.029	0.039	0.031
$(\sin \theta/\lambda)_{max}$ (Å^{-1})	0.625	0.595	0.596	0.612
Refinement				
$R[F^2 > 2\sigma(F^2)]$, $wR(F^2)$, S	0.026, 0.067, 1.15	0.027, 0.068, 1.16	0.057, 0.148, 1.15	0.028, 0.075, 1.23
No. of parameters	22	22	22	22
No. of restraints	13	13	13	13
Weighting scheme	$w = 1/[\sigma^2(F_o^2) + (0.040P)^2 + 1.4758P]$ where $P = (F_o^2 + 2F_c^2)/3$	$w = 1/[\sigma^2(F_o^2) + (0.0462P)^2 + 1.030P]$ where $P = (F_o^2 + 2F_c^2)/3$	$w = 1/[\sigma^2(F_o^2) + (0.1035P)^2 + 6.7505P]$ where $P = (F_o^2 + 2F_c^2)/3$	$w = 1/[\sigma^2(F_o^2) + (0.0528P)^2 + 0.427P]$ where $P = (F_o^2 + 2F_c^2)/3$
$\Delta\rho_{max}$, $\Delta\rho_{min}$ (e Å^{-3})	0.50, −0.34	0.52, −0.49	0.90, −1.10	0.39, −0.73

Table S4. *Cont.*

Pressure (GPa)	2.27	2.57	2.92	3.87
Crystal data				
Crystal system, space group	Monoclinic, *I*2/*a*	Monoclinic, *I*2/*a*	Monoclinic, *I*2/*a*	Monoclinic, *I*2/*a*
a, *b*, *c* (Å)	7.1432 (3), 10.3793 (5), 8.4049 (15)	7.0643 (16), 10.364 (6), 8.305 (5)	7.0483 (3), 10.3431 (5), 8.3088 (16)	6.9441 (4), 10.2934 (6), 8.224 (2)
α, β, γ (°)	90, 102.112 (5), 90	90, 102.070 (16), 90	90, 101.683 (6), 90	90, 101.200 (8), 90
V (Å^3)	609.28 (12)	594.6 (5)	593.17 (12)	576.61 (16)
Z	4	4	4	4
Radiation type	Mo $K\alpha$	Synchrotron, λ = 0.7749 Å	Mo $K\alpha$	Mo $K\alpha$
μ (mm^{-1})	5.14	6.56	5.28	5.43
Crystal size (mm)	0.20 × 0.10 × 0.10	0.22 × 0.18 × 0.12	0.20 × 0.10 × 0.10	0.20 × 0.10 × 0.10
Data collection				
Diffractometer	Bruker *APEX* 2	Bruker D8 with Photon II detector	Bruker *APEX* 2	Bruker *APEX* 2
T_{min}, T_{max}	0.531, 0.745	0.546, 0.745	0.550, 0.745	0.525, 0.745
No. of measured, independent and observed [$I > 2\sigma(I)$] reflections	1130, 214, 206	368, 227, 225	1110, 204, 199	1061, 195, 194
R_{int}	0.030	0.014	0.028	0.037
$(\sin \theta/\lambda)_{max}$ (Å^{-1})	0.625	0.592	0.620	0.622
Refinement				
$R[F^2 > 2\sigma(F^2)]$, $wR(F^2)$, S	0.026, 0.072, 1.19	0.045, 0.115, 1.13	0.025, 0.071, 1.26	0.026, 0.073, 1.17
No. of parameters	22	22	22	22
No. of restraints	13	13	13	12
Weighting scheme	$w = 1/[\sigma^2(F_o^2) + (0.0423P)^2 + 3.6529P]$ where $P = (F_o^2 + 2F_c^2)/3$	$w = 1/[\sigma^2(F_o^2) + (0.0946P)^2 + 2.1155P]$ where $P = (F_o^2 + 2F_c^2)/3$	$w = 1/[\sigma^2(F_o^2) + (0.0329P)^2 + 5.1882P]$ where $P = (F_o^2 + 2F_c^2)/3$	$w = 1/[\sigma^2(F_o^2) + (0.0454P)^2 + 2.8929P]$ where $P = (F_o^2 + 2F_c^2)/3$
$\Delta\rho_{max}$, $\Delta\rho_{min}$ (e Å^{-3})	0.44, −0.68	0.76, −0.89	0.46, −0.50	0.33, −0.56

Table S4. *Cont.*

Pressure (GPa)	4.02	4.51	4.93	5.00
Crystal data				
Crystal system, space group	Monoclinic, *I*2/*a*	Monoclinic, *I*2/*a*	Monoclinic, *I*2/*a*	Monoclinic, *I*2/*a*
a, *b*, *c* (Å)	6.9284 (18), 10.246 (5), 8.209 (6)	6.8808 (4), 10.2680 (6), 8.146 (2)	6.849 (3), 10.207 (7), 8.119 (9)	6.8316 (3), 10.2395 (6), 8.0931 (18)
α, β, γ (°)	90, 100.967 (18), 90	90, 100.907 (8), 90	90, 100.87 (3), 90	90, 100.696 (7), 90
V ($Å^3$)	572.1 (5)	565.12 (15)	557.3 (8)	556.29 (13)
Z	4	4	4	4
Radiation type	Synchrotron, λ = 0.7749 Å	Mo *K*α	Synchrotron, λ = 0.7749 Å	Mo *K*α
μ (mm^{-1})	6.82	5.54	7.00	5.63
Crystal size (mm)	0.22 × 0.18 × 0.01	0.20 × 0.10 × 0.10	0.22 × 0.18 × 0.12	0.20 × 0.10 × 0.10
Data collection				
Diffractometer	Bruker D8 with Photon II detector	Bruker *APEX* 2	Bruker D8 with Photon II detector	Bruker *APEX* 2
T_{min}, T_{max}	0.530, 0.745	0.605, 0.745	0.430, 0.745	0.601, 0.745
No. of measured, independent and observed [$I > 2\sigma(I)$] reflections	508, 233, 220	1054, 198, 195	457, 227, 218	1028, 192, 189
R_{int}	0.042	0.034	0.039	0.039
$(\sin \theta/\lambda)_{max}$ ($Å^{-1}$)	0.594	0.625	0.596	0.622
Refinement				
$R[F^2 > 2\sigma(F^2)]$, $wR(F^2)$, S	0.050, 0.128, 1.17	0.039, 0.104, 1.12	0.055, 0.131, 1.17	0.043, 0.114, 1.19
No. of parameters	22	22	22	22
No. of restraints	13	13	13	13
Weighting scheme	$w = 1/[\sigma^2(F_o^2) + (0.0973P)^2]$ where $P = (F_o^2 + 2F_c^2)/3$	$w = 1/[\sigma^2(F_o^2) + (0.069P)^2 + 6.2397P]$ where $P = (F_o^2 + 2F_c^2)/3$	$w = 1/[\sigma^2(F_o^2) + (0.0988P)^2]$ where $P = (F_o^2 + 2F_c^2)/3$	$w = 1/[\sigma^2(F_o^2) + (0.0676P)^2 + 8.0187P]$ where $P = (F_o^2 + 2F_c^2)/3$
$\Delta\rho_{max}$, $\Delta\rho_{min}$ (e $Å^{-3}$)	1.19, −1.10	0.81, −0.56	1.03, −0.86	1.19, −0.56

Table S4. *Cont.*

Pressure (GPa)	5.49	8.14
Crystal data		
Crystal system, space group	Triclinic, *P*−1	Triclinic, *P*−1
a, *b*, *c* (Å)	5.692 (5), 6.9674 (11), 7.7283 (12)	5.586 (3), 6.876 (5), 7.699 (2)
α, β, γ (°)	65.412 (13), 80.98 (4), 87.77 (3)	64.93 (3), 79.93 (3), 86.84 (5)
V (Å^3)	275.1 (3)	263.6 (2)
Z	2	2
Radiation type	Synchrotron, λ = 0.7749 Å	Mo $K\alpha$
μ (mm^{-1})	7.09	5.94
Crystal size (mm)	0.1 × 0.1 × 0.1	0.05 × 0.05 × 0.05
Data collection		
Diffractometer	Bruker D8 with Photon II detector	Bruker *APEX* II
T_{min}, T_{max}	0.390, 0.745	0.549, 0.745
No. of measured, independent and observed [$I > 2\sigma(I)$] reflections	549, 236, 197	586, 246, 211
R_{int}	0.041	0.061
(sin θ/λ)$_{max}$ (Å^{-1})	0.575	0.625
Refinement		
$R[F^2 > 2\sigma(F^2)]$, $wR(F^2)$, S	0.111, 0.293, 1.34	0.059, 0.142, 1.22
No. of parameters	30	30
No. of restraints	34	34
Weighting scheme	$w = 1/[\sigma^2(F_o^2) + (0.2P)^2]$ where $P = (F_o^2 + 2F_c^2)/3$	$w = 1/[\sigma^2(F_o^2) + (0.0402P)^2 + 13.5677P]$ where $P = (F_o^2 + 2F_c^2)/3$
$\Delta\rho_{max}$, $\Delta\rho_{min}$ (e Å^{-3})	1.87, −1.16	0.81, −0.79

4. References

1. Chickos, J.S.; Jr., W.E.A. Enthalpies of sublimation of organic and organometallic compounds. 1910–2001. *Journal of Physical and Chemical Reference Data* **2002**, *31*, 537–698.
2. Groom, C.R.; Bruno, I.J.; Lightfoot, M.P.; Ward, S.C. The cambridge structural database. *Acta Crystallographica Section B* **2016**, *72*, 171–179.
3. Gavezzotti, A. *Calculation of lattice energies of organic crystals: The pixel integration method in comparison with more traditional methods*. 2005; Vol. 220, p 499–510.
4. Clark, S.J.; Segall, M.D.; Pickard, C.J.; Hasnip, P.J.; Probert, M.J.; Refson, K.; Payne, M.C. First principles methods using castep. *Zeitschrift für Kristallographie* **2005**, *220*, 567–570.
5. Gavezzotti, A. *Molecular aggregation - structure analysis and molecular simulation of crystals and liquids*. 1st ed.; Oxford University Press: New York, 2007; p 425.
